# Epidemiological, Socioeconomic, and Health Service Factors Associated with Tuberculosis Treatment Interruption in Brazil

**DOI:** 10.3390/epidemiologia6040081

**Published:** 2025-11-26

**Authors:** Jéssica Simões Mendonça, Fabrício Sette Abrantes Silveira, Renata Maria Colodette, Deíse Moura de Oliveira, Érica Toledo de Mendonça, Rosângela Minardi Mitre Cotta, Antônio Almeida de Barros Junior, João Vitor Andrade, Tiago Ricardo Moreira

**Affiliations:** 1 Department of Medicine and Nursing, Federal University of Viçosa, Av. Peter Henry Rolfs, University Campus, Viçosa 36570-900, MG, Brazil; jessica.s.mendonca@ufv.br (J.S.M.); fabricio.sette@ufv.br (F.S.A.S.); deise.oliveira@ufv.br (D.M.d.O.); erica.mendonca@ufv.br (É.T.d.M.); 2 University Center of Viçosa, Av. Maria de Paula Santana, 3815 Silvestre, Viçosa 36576-340, MG, Brazil; renatacolodette@yahoo.com.br; 3 Department of Health Nutrition, Federal University of Viçosa, Av. Peter Henry Rolfs, University Campus, Viçosa 36570-900, MG, Brazil; rmmitre@ufv.br; 4 Department of Computing, Center for Exact, Natural, and Health Sciences, Federal University of Espírito Santo, Alto Universitário, P.O. Box 16 Guararema, Alegre 29500-000, ES, Brazil; antonio.barros@ufes.br; 5 School of Nursing, Federal University of Alfenas, R. Gabriel Monteiro da Silva, 700 Centro, Alfenas 37130-001, MG, Brazil; jvma100@gmail.com

**Keywords:** tuberculosis, pulmonary, medication adherence, epidemiology, health management, social determinants of health

## Abstract

**Background**: Brazil must make progress toward eliminating tuberculosis as a public health problem and achieving the goal of reducing treatment interruption to below 5%. Improving adherence requires a thorough understanding of the factors that influence this outcome. **Objectives**: To identify epidemiological, socioeconomic, and health service-related factors associated with tuberculosis treatment interruption in Brazilian municipalities from 2018 to 2022. **Methods**: This ecological study utilized secondary data from all Brazilian municipalities. Independent variables were organized into three blocks: epidemiological, health service coverage, and socioeconomic. A zero-inflated beta regression model was employed to analyze both the proportion and zero-inflated components. **Results**: The mean treatment interruption rate was 8.1%. Interruption was associated with the proportion of laboratory-confirmed cases, Family Health Strategy coverage, and the proportion of the population residing in rural areas. Tuberculosis incidence, sputum smear microscopy, molecular rapid tests, contact investigation, directly observed therapy, AIDS detection rate, Gini index, household crowding, and illiteracy were associated with treatment adherence. In the zero-inflated component, directly observed therapy, consultations per inhabitant, illiteracy, and the proportion of the population residing in rural areas increased the probability of a zero-interruption rate, whereas TB incidence, AIDS detection, municipal population, and household crowding decreased that probability. **Conclusions**: Tuberculosis treatment interruption in Brazil is shaped by socioeconomic, epidemiological, and health service factors, highlighting the need for integrated strategies that combine social protection with strengthened primary care to improve adherence and progress toward elimination goals.

## 1. Introduction

In 2023, with COVID-19 (Coronavirus Disease 2019) no longer classified as a global public health emergency, tuberculosis (TB) has likely resumed its position as the leading cause of death from a single infectious agent [1]. According to estimates released by the World Health Organization (WHO), the COVID-19 pandemic in 2020 resulted in a 25% decrease in TB diagnoses and a 26% increase in mortality worldwide. Additionally, it exacerbated the ongoing health and social crisis in most countries where the disease remains a public health concern, thereby intensifying the challenges of addressing TB, a disease strongly influenced by social determinants that contribute to the persistence of inequalities [2].

In Brazil, tuberculosis treatment is provided free of charge and universally through the Unified Health System (Sistema Único de Saúde—SUS), which serves as the sole pathway for accessing medications. Despite this guaranteed provision, the prolonged duration of therapy remains a challenge to adherence, as clinical improvement often occurs shortly after treatment initiation, leading some patients to discontinue medication prematurely. The standard regimen for drug-susceptible pulmonary tuberculosis includes a combination of up to four antibiotics, administered daily until treatment completion [3]. For individuals aged 17 years and older, the minimum duration is six months, which may be extended to nine months depending on clinical progression. In contrast, for children and adolescents between 3 months and 16 years of age, treatment duration may be reduced to four months [4].

Recent national indicators of tuberculosis treatment performance demonstrate a progressive decline in cure rates for new, laboratory-confirmed cases: 72.1% in 2020, 70.5% in 2021, 69.4% in 2022, and 66% in 2023. Over the same period, treatment interruption rates were 14%, 14.8%, 15.2% and 15.3%, respectively [5]. These results stand in marked contrast to the benchmarks recommended by the World Health Organization (WHO), which define global targets as cure rates above 85% and treatment default rates below 5% [6,7]. Given that tuberculosis is transmitted via the respiratory route and that individuals who fail to complete treatment remain potential sources of infection, not achieving these targets compromises long-term reductions in incidence rates [3,6,7].

An already unfavorable epidemiological scenario has been further exacerbated by the COVID-19 pandemic. Therefore, advancing toward the elimination of TB as a public health problem requires an understanding of how the multiple factors associated with the disease’s manifestation may influence case outcomes.

Analyzing the factors associated with treatment interruption through an ecological study allows for the identification of how health interventions, the socioeconomic context, and healthcare services contribute to treatment interruption. The findings may inform the development of more effective public policies that address the specific needs of local territories.

Therefore, this study aims to identify the socioeconomic, epidemiological, and operational health factors associated with TB treatment interruption in Brazilian municipalities from 2018 to 2022.

## 2. Materials and Methods

### 2.1. Study Design and Population Base

This ecological study used Brazilian municipalities as units of analysis. All municipalities that had reported at least one case of tuberculosis to the Notifiable Diseases Information System (Sinan) between 2018 and 2022 were included. Only municipalities with at least one new case of pulmonary tuberculosis were retained, while those with treatment outcomes recorded as blank or ignored were excluded.

### 2.2. Data Sources and Variables

Data were obtained from ten publicly accessible online databases, all of which were freely available. These sources included socioeconomic indicators, TB epidemiological data, and health service coverage variables. The databases consulted were: the Department of Informatics of the Unified Health System (DATASUS); the Department of HIV/AIDS, Tuberculosis, Viral Hepatitis, and Sexually Transmitted Infections (DATHI); the Primary Health Care Management Portal (e-Gestor APS); the Brazilian Institute of Geography and Statistics (IBGE); and the Human Development Atlas in Brazil (Atlas Brasil).

The dependent variable was the proportion of treatment interruption among new pulmonary TB cases, calculated as the number of new pulmonary TB cases classified as treatment interruption from 2018 to 2022 divided by the total number of new pulmonary TB cases reported during the same period, multiplied by 100.

Independent variables were selected based on previous studies that had identified them as influential factors in TB outcomes [8,9,10]. These were organized into three blocks: (1) epidemiological and operational variables related to the Tuberculosis Control Program; (2) health service coverage variables; and (3) socioeconomic variables.

To mitigate potential bias arising from variability in small municipalities and inconsistencies in reporting, variables related to the TB Control Program were aggregated over the period from 2018 to 2022. Socioeconomic data corresponded to the most recent year available in each database. To minimize the potential impact of the COVID-19 pandemic, variables related to health service coverage and AIDS case detection rates were taken exclusively from 2019. A complete description of all independent variables is provided in Table 1.

### 2.3. Data Collection and Statistical Analysis Procedures

Data collection occurred between March and August 2024. The data were tabulated using Microsoft Office^®^ Excel and the Statistical Package for Social Sciences (SPSS 22.0^®^), and processed in R software version 4.3.0 (R Core Team, 2023). All statistical analyses were conducted with 95% confidence intervals (CI 95%).

A descriptive analysis was initially performed using means, standard deviations, medians, and interquartile ranges.

### 2.4. Spatial Analysis

To assess spatial autocorrelation in the data, municipalities were converted into spatial objects using geographic coordinates (latitude and longitude), then projected onto the UTM reference system (SIRGAS 2000, zone 23S). A spatial neighborhood matrix was constructed based on a maximum distance of 50 km between centroids (dnearneigh), which was then transformed into a spatial weights matrix (listw) with row-standardized weighting (style W).

Global spatial autocorrelation was evaluated using Moran’s I test applied to both the dependent variable and residuals of the fitted models. Upon detection of significant spatial autocorrelation in the initial model residuals, a two-dimensional spatial smoothing term using penalized splines (pb(X), pb(Y) gamlss package) was added to the municipalities’ geographic coordinates to control for unexplained spatial dependence. Smoothing was performed automatically using the RS [11] method, which iteratively adjusts penalty parameters to balance model complexity and goodness of fit.

The Local Moran’s I (Local Indicators of Spatial Association—LISA) was then estimated for each municipality, which allowed the identification of areas with significant patterns of positive spatial association (High–High and Low–Low clusters) or negative spatial association (High–Low and Low–High clusters). Municipalities with no statistical significance (*p* > 0.05) were classified as “not significant.”

For cartographic representation, municipalities were categorized according to the cluster typology obtained, and the results were displayed in thematic maps (LISA maps), with state and national boundaries delineated to facilitate regional interpretation.

### 2.5. Statistical Modeling

Given the concentration of municipalities with 0% treatment interruption, a beta zero-inflated (BEZI) regression model was employed to identify factors associated with treatment interruption among new pulmonary TB cases.

For model fitting, the dependent variable was adjusted: values equal to 1 were replaced with 0.9999, as the beta distribution does not accommodate exact ones in its continuous component. Zero values were retained, since they are addressed by the model’s zero-inflated component. The robustness of this transformation was assessed through sensitivity analyses, in which the value of 1 was replaced with 0.999 and, alternatively, municipalities with a proportion equal to 1 (0.94% of the sample) were excluded. These procedures yielded no material changes in the estimates.

The regression was estimated using the gamlss package in R, which enabled simultaneous modeling of: (i) the continuous component of the response variable (restricted to the 0–1 interval) through the μ parameter, using a logit link function; and (ii) the zero-inflated component through the ν parameter, also with a logit link, representing the probability that the proportion of treatment interruption was exactly zero (i.e., no treatment interruption in the municipality).

Subsequently, a univariate analysis was performed to examine the association between each variable block and TB treatment interruption. Variables with *p* > 0.200 were excluded. Remaining variables were ranked by influence using the Wald test, and strongly correlated variables (Spearman’s r ≥ 0.6) were screened; among these, only the most influential according to the Wald test were retained for the multivariate model.

In the multivariate regression, distinct explanatory variables were incorporated into the μ and ν equations to assess both the intensity of treatment interruption and its complete absence. A hierarchical block-wise modeling strategy was employed: the first block included epidemiological variables, the second health service coverage, and the third on socioeconomic variables. Variables with *p* < 0.05 were retained in each model. Associations were evaluated using regression coefficients and their 95% confidence intervals.

### 2.6. Model Evaluation

Model performance was assessed using global deviance, the Akaike Information Criterion (AIC), and residual diagnostics. The absence of spatial autocorrelation in the residuals was verified with a subsequent Moran’s I test. All modeling procedures were carried out in R (version 4.3 or later) using the gamlss, sf, spdep, dplyr, and other supporting packages.

### 2.7. Ethical Considerations

This study used aggregated, publicly available data without names or personal identifiers and therefore did not require approval from a Research Ethics Committee [12].

## 3. Results

Between 2018 and 2022, a total of 472,781 TB cases were diagnosed across 5301 Brazilian municipalities. Of these, 374,476 were pulmonary TB cases, reported in 5223 municipalities.

After selecting new pulmonary TB cases and excluding those with missing or unclassified treatment outcomes, 5007 municipalities were eligible for analysis (loss of 4.14%). Among these, 47.27% (*n* = 2367) had treatment interruption rates different from 0% and 100%; 51.79% (*n* = 2593) had a 0% interruption rate; and 0.94% (*n* = 47) had a 100% treatment interruption rate.

The mean treatment interruption rate in Brazil during the study period was 8.10%, ranging from 0.00% to 12.00%.

The incidence of pulmonary TB varied widely among municipalities, with a mean of 92.251 new cases and a median of 67.760 (IQR 40.040 to 107.840). Family Health Strategy (FHS) coverage was high in most municipalities, with an average of 88.25% and a median of 100% (IQR 84.99% to 100%). Social inequality, measured by the Gini Index, had a mean of 0.50 and a median of 0.50 (IQR 0.45 to 0.54), indicating a scenario of moderate socioeconomic disparity. Additional results are presented in Table 2.

In the univariate analysis, the following variables were positively associated with TB treatment interruption: TB-HIV coinfection rate, number of consultations per capita per year, vaccine coverage homogeneity, FHS and CHA coverage, MHDI, life expectancy at birth, and the proportion of the population residing in rural areas. Conversely, the following variables were inversely associated: incidence and mortality rates, performance of sputum smear microscopy, rapid molecular TB testing (TRM-TB), drug susceptibility testing, HIV testing, cases among vulnerable groups, proportion of contacts examined, directly observed therapy (DOT) rate, AIDS detection rate, Gini index, unemployment, household overcrowding, illiteracy, and proportion of the population living in poverty.

In the zero-inflated component, the following variables were inversely associated with the probability of a 0% treatment interruption rate in the univariate analysis: incidence and mortality rates, drug susceptibility testing, HIV testing, proportion of laboratory-confirmed cases, TB-HIV coinfection, cases among vulnerable groups, AIDS detection rate, SUS hospital beds, population size, population density, Gini index, MHDI, family income, unemployment, household overcrowding, and life expectancy at birth. Conversely, the following variables were positively associated with the probability of a 0% treatment interruption rate: DOT, number of consultations per capita per year, FHS and CHA coverage, illiteracy, proportion of the population living in poverty, and proportion of the population residing in rural areas, according to Table 3.

The variables Municipal Human Development Index (MHDI), per capita GDP, life expectancy at birth, proportion of the population living in poverty, and Community Health Agent (CHA) coverage were not included in the multivariate models due to strong correlations with other variables that demonstrated greater associations with the dependent variable. The infant mortality rate was also excluded from the adjusted models because it did not show a significant association with TB treatment interruption.

Table 4 presents the associations between socioeconomic, epidemiological, and operational health variables and TB treatment interruption for each model. In Model 1 (epidemiological and operational variables related to the TB Control Program), most variables remained significantly associated with TB treatment interruption, except for TB mortality rate and the proportion of cases among vulnerable groups, which lost significance.

With the inclusion of health service coverage variables in Model 2, only the proportion of cases tested for HIV lost significance. All other health service coverage variables remained statistically significant, except for CHA coverage.

In Model 3, after adding socioeconomic variables, the following lost statistical significance: TB-HIV coinfection, number of SUS beds, vaccine coverage homogeneity, population density, and unemployment rate.

The inclusion of geographic variables in Model 4 identified the following associations with TB treatment interruption and adherence for each block:

Block 1—Epidemiological Variables: Treatment interruption was associated with the proportion of laboratory-confirmed cases. Conversely, TB incidence, performance of diagnostic tests (sputum smear microscopy and TRM-TB), proportion of contacts examined, DOT, and AIDS detection were associated with treatment adherence. In the zero-inflated component, DOT increased the likelihood of zero treatment interruption, whereas TB incidence and AIDS detection decreased this likelihood.

Block 2—Health Service Coverage Variables: Treatment interruption was associated with FHS coverage. In the zero-inflated component, the number of consultations per capita increased the probability of zero treatment interruption.

Block 3—Socioeconomic Variables: The Gini index, illiteracy rate, and household overcrowding were associated with treatment adherence. The proportion of the population residing in rural areas was associated with treatment interruption. In the zero-inflated component, both the proportion of the population residing in rural areas and the illiteracy rate increased the probability of zero treatment interruption, whereas population size and household overcrowding decreased this probability.

The final model included data from 4782 municipalities, yielding a global deviance of 1035.138 (AIC: 1122.579). Spatial autocorrelation of the dependent variable (treatment interruption rate for TB) was assessed using Moran’s I. Prior to the inclusion of smoothing terms, significant positive autocorrelation was observed (Moran’s I = 0.0432; *p* < 0.001), indicating spatial clustering of treatment interruption. After fitting the unsmoothed model, Moran’s I decreased to 0.0330 (*p* < 0.001), whereas the inclusion of penalized spline terms increased Moran’s I to 0.0527 (*p* < 0.001). These results suggest that penalized splines capture part of the spatial dependence, although subtle residual patterns remain.

**Figure 1:**
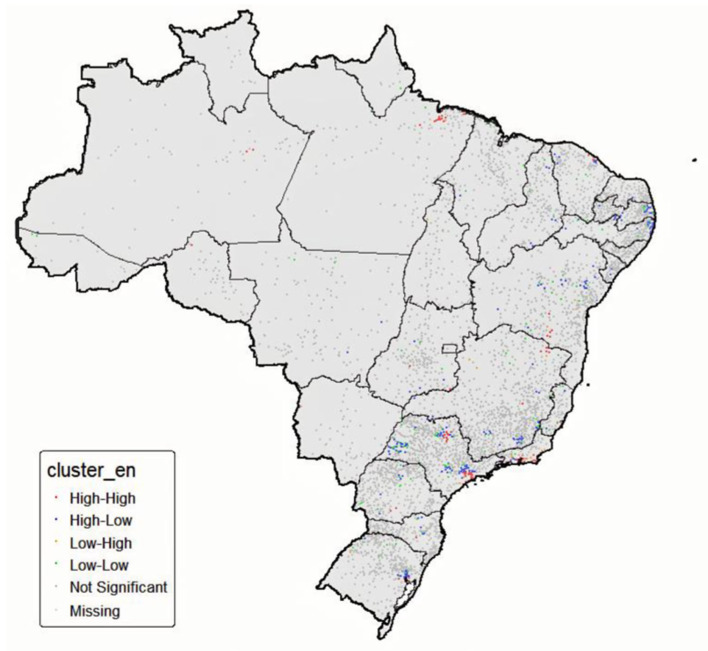
Local spatial clusters (LISA) of residuals from the final model of tuberculosis treatment interruption in Brazilian municipalities, 2018–2022.

The spatial analysis further demonstrated that additive terms for longitude (pb(X)) remained significant in the proportion component (μ), indicating a residual East–West gradient, while latitude (pb(Y)) had no detectable effect. To further explore this pattern, Local Moran’s I (LISA) was applied to the residuals of the final model. The resulting map (Figure 1) revealed “High–High” clusters predominantly in the North and Central-West regions of Brazil, reflecting municipalities with elevated residual treatment interruption values adjacent to one another. Conversely, “Low–Low” clusters were concentrated in the South and Southeast, suggesting spatial groupings of lower residual values. Taken together, these findings indicate that, despite adjustment for epidemiological, sociodemographic, and healthcare service variables, regional heterogeneity persists and warrants consideration in tuberculosis control policies.

## 4. Discussion

The presence of significant coefficients across all three blocks of variables indicates that factors related to the Tuberculosis Control Program, health services, and socioeconomic characteristics can all influence treatment interruption.

The complexity of the analytical approach, which evaluates TB treatment interruption through both components of the model (proportion and zero-inflated), may occasionally produce findings that appear contradictory. However, this apparent contradiction arises from the interpretative nature of the two components of the zero-inflated beta regression model. Therefore, it is important to understand that these results are not conflicting but complementary, reflecting the complex relationships between the variables and the outcome.

The results show that, depending on the model component, the analyzed variables may have different impacts on TB treatment interruption. An inverse association in the proportion component indicates that an increase in the independent variable reduces the proportion of the dependent variable, whereas in the zero-inflated component, an inverse association implies that an increase in the independent variable decreases the probability that the dependent variable equals zero.

The analysis revealed that tuberculosis incidence exhibits distinct associations with the components of the treatment interruption model. In the proportion component, higher incidence was associated with reduced treatment interruption. Conversely, in the zero-inflation component, a negative coefficient indicated that higher incidence was linked to an increased probability of treatment interruption. These contrasting relationships may be explained by the spatial heterogeneity of the disease, which has been widely documented in diverse geographic contexts across Brazil and other Latin American countries [13,14,15]. In high-incidence areas, better access to healthcare services may contribute to lower treatment interruption rates [8,16,17], whereas in low-incidence areas, limited access may increase the treatment interruption rates [18,19].

The performance of sputum smear microscopy and rapid molecular testing for TB (TRM-TB) was found to be associated with reduced treatment interruption. Some studies suggest that the absence of smear microscopy and other diagnostic and monitoring procedures may contribute to higher treatment interruption rates [20,21]. In our review, we did not identify studies directly evaluating the association between TRM-TB and treatment interruption, likely because TRM-TB is relatively recent compared with smear microscopy and remains underutilized by healthcare professionals, as reflected in our findings.

TRM-TB, based on the Xpert MTB/RIF technology, has been progressively adopted in low- and middle-income countries; however, its implementation faces substantial operational barriers, including limited machine availability, maintenance costs, the need for workforce training, and logistical challenges related to specimen transport [22,23]. Moreover, multicenter studies have shown that even after the introduction of TRM-TB, diagnostic coverage and its impact on tuberculosis indicators may vary widely depending on installed capacity and local healthcare conditions [24,25].

In Brazil, the Rapid Molecular Testing Network for Tuberculosis was introduced into the Unified Health System (SUS) in 2014. By 2022, a total of 257 machines were available, distributed across 135 municipalities in all states of the Federation [26]. Considering the country’s territorial extension, this number remains insufficient to ensure full nationwide coverage, particularly in light of the logistical constraints already described [27]. Accordingly, the low mean utilization observed (27%) should be interpreted in the context of these implementation barriers, which helps contextualize our findings and underscores the need for strategies aimed not only at network expansion but also at improving operational performance.

In contrast to the above results, the proportion of new cases confirmed through laboratory testing was associated with an increase in the proportion of TB treatment interruption in municipalities. Unlike culture and drug susceptibility testing, bacilloscopy and TRM-TB are diagnostic tools that provide rapid results, enabling treatment initiation within a few days. This may enhance confidence in the diagnosis and reflect stronger integration between local health services and the community. Moreover, although drug susceptibility testing is recommended for all confirmed TB cases [28], our results suggest that it remains underutilized by healthcare professionals.

Contact tracing for TB, followed by assessment to identify active disease and/or latent infection, not only contributes to the diagnosis of new cases but also helps reduce follow-up loss and unfavorable outcomes, including treatment interruption [29,30]. In this context, a higher proportion of contacts examined may indirectly reflect improved TB surveillance, as this activity entails home visits, family counseling, and regular follow-up, factors that, together, may contribute to improved treatment outcomes at the municipal level.

Similarly, in both the proportion component and the zero-inflated component, DOT (directly observed therapy) was associated with a reduction and even the absence of treatment interruption, indicating that this strategy contributes to more favorable TB outcomes in municipalities. Several studies, using initial data, have emphasized that treatment interruption is less prevalent among individuals who received DOT, while treatment discontinuation is more frequent among those who underwent self-administered treatment [8,31,32]. However, a study using data from a municipality in the state of Mato Grosso associated DOT with treatment dropout [23], highlighting the importance of states and municipalities assessing their needs according to local realities.

The AIDS detection rate was associated with both a reduction in the proportion of treatment interruption and a reduction in the probability of having zero interruption. This finding may relate to TB-HIV coinfection cases, as most studies examining factors contributing to treatment discontinuation cite TB-HIV association as a key factor in non-adherence [8,31,32,33,34,35]. Moreover, people living with HIV/AIDS (PLWHA) are 15–21 times more likely to progress to active TB compared to the general population. Although treatment interruption rates are higher among PLWHA compared to individuals without coinfection, it is possible that, in certain contexts, these patients may have a better understanding of the importance of TB treatment adherence due to their experience with regular clinical monitoring related to their HIV status. Therefore, it is important to consider that conditions leading to TB and HIV exposure and illness may be shaped by inequities linked to local health system contexts, social, economic, behavioral, and cultural disparities, as well as healthcare service organization [36].

Among less intuitive findings, increased FHS coverage was associated with higher treatment interruption rates. This may suggest structural problems or failures in adherence. Despite expanded access, there may be challenges in the effectiveness of care provided, as some studies have pointed to the organization of local health services, the decentralization of treatment to FHSs, and the engagement of professionals as facilitators or hindrances to treatment continuity at the municipal level. An integrative review published in 2024 reported that the quality of care provided by health units contributes to treatment interruption [37]. Another literature review examining studies from 2000 to 2009 linked treatment interruption to poor interaction and communication between patients and healthcare professionals [34].

These apparently contradictory findings—with DOT being protective while higher FHS coverage was associated with treatment interruption—warrant cautious interpretation. Since the implementation of DOT is usually the responsibility of FHS teams, it is possible that contextual factors such as organizational capacity, workload, or heterogeneity in how DOT is delivered across municipalities may explain these divergent associations.

Expanding coverage and improving health infrastructure are essential, but these efforts must be accompanied by effective strategies that promote treatment adherence and support the decentralization of care to primary healthcare (PHC) units. This strengthens their role as the preferred point of entry into the healthcare system, thereby improving access to services. Another key issue is the need for ongoing training of PHC teams on TB-related issues, so they are equipped to identify new cases and effectively monitor patients undergoing treatment [38,39].

In the zero-inflated component, an increased number of medical consultations per capita was associated with a higher probability of zero treatment interruption, indicating that easier access to health services and resources supports treatment continuity [21,33,35].

Municipal population size, which was inversely associated with the probability of having zero treatment interruption, may reflect difficulties in meeting all healthcare demands. A descriptive study based on a narrative review of Brazilian studies published between 2016 and 2021 evaluated concepts and factors associated with TB treatment non-adherence in PHC and identified high service demand as a contributing factor [35]. An ecological study currently in publication, using data from individuals residing in the state of São Paulo diagnosed with TB between January 2012 and December 2017, found that individuals living in medium- and large-sized municipalities had 2.64 (95% CI: 2.15–3.24) and 4.48 (95% CI: 4.62–5.55) times greater risk, respectively, of abandoning TB treatment compared to those living in small municipalities [40].

In the present study, the proportion of the population residing in rural areas exhibited a dual relationship with tuberculosis treatment default. Municipalities with a higher share of rural residents were more likely not to report treatment interruption—possibly due to the low absolute incidence of cases, a feature of sparsely populated and dispersed areas. However, in municipalities where treatment default did occur, its proportion was significantly higher, reflecting structural barriers such as geographic inaccessibility, limited health resources, and reduced availability of diagnostic and follow-up services—factors commonly observed in rural contexts distant from urban centers [41,42].

These findings reinforce the existing literature that identifies rural settings as a marker of territorial vulnerability: distance from health services and transportation costs may hinder treatment regularity even in the presence of the Family Health Strategy [43]. Thus, the proportion of rural population functions as an indicator of spatial inequality, highlighting that although many rural municipalities may not formally register treatment default, in those that do, treatment continuity tends to be fragile.

The Gini index, which assesses income distribution in municipalities, was associated with a reduction in treatment interruption. A cohort study conducted in the state of Sergipe, which characterized TB treatment interruption between 2015 and 2018, found that individuals experiencing homelessness or living in municipalities with high MHDI and high income inequality had a higher likelihood of treatment interruption [44]. Considering the size and diversity of the country, these results may require further investigation, as they may reflect regional variations.

Household overcrowding showed contradictory results, being associated both with a reduction in treatment interruption and a reduction in the probability of the interruption rate being zero. This reflects both protective collective aspects and social vulnerability. As several studies have described, household overcrowding not only contributes to the continued transmission of the disease but may also reflect socioeconomic vulnerabilities that increase the risk of TB treatment interruption [35,37,45,46].

However, large families may provide emotional support to the patient, facilitating treatment follow-up, since the absence of a support network and conflictual family relationships may hinder treatment adherence [34,35]. In addition, it is important to highlight that receiving benefits and/or incentives (such as basic food baskets and transportation vouchers) may play a significant role in supporting treatment adherence and in strengthening the relationship between the patient and the healthcare team [47,48].

Higher municipal illiteracy rates were associated with a lower proportion of treatment interruption, potentially reflecting the prioritization of Primary Health Care and the Family Health Strategy in more vulnerable areas, which may facilitate treatment adherence [16,17,49,50]. In the zero-inflation component, illiteracy was associated with a greater likelihood of zero interruptions, an effect that could be related to the low absolute number of cases in small and rural municipalities, as well as the inherent limitations of the ecological design, which precludes individual-level inference [51,52].

Certain variables, including TB-HIV coinfection, homogeneity of vaccination coverage, and the number of SUS hospital beds per 1000 inhabitants, were significant in initial models but lost relevance following the inclusion of socioeconomic and demographic covariates, and were subsequently excluded from the final model. This shift suggests that part of the effect of these variables is shared with other determinants, underscoring the importance of simultaneously considering multiple blocks of factors. Nevertheless, the health services block retained explanatory significance, as indicators such as the number of medical consultations per inhabitant per year and the coverage rate of the Family Health Strategy (FHS) remained associated with the outcome.

This study has several limitations that warrant consideration.

First, the ecological design is subject to the so-called ecological fallacy, which occurs when associations observed at the aggregate level are interpreted as valid at the individual level [51,52]. Therefore, the results should be interpreted at the municipal level and cannot be directly extrapolated to individuals. Nevertheless, the findings provide relevant insights for understanding population-level patterns and informing public health policies.

Moreover, the findings are closely tied to the organizational and epidemiological characteristics of the Brazilian Unified Health System (SUS), which limits their direct applicability to other health systems. At the same time, Brazil’s continental dimensions, marked by socioeconomic, cultural, and geographic heterogeneity, together with the large dataset and robust analytical approach employed, support the relevance of the methodological framework for studies in other settings with comparable complexities.

A further constraint relates to the use of secondary data, whose quality depends on the accurate completion of notification forms by healthcare professionals, potentially leading to inconsistencies relative to the original records.

In addition, some socioeconomic indicators were derived from the 2010 Demographic Census, which may introduce temporal bias given that Brazil has experienced substantial socioeconomic changes over the past decade. This dataset was selected, however, as it encompasses all 5007 municipalities analyzed, ensuring national comparability and methodological consistency. Whenever possible, more recent variables, such as those from the 2022 Census, were incorporated to mitigate this temporal gap.

The study was also limited by the absence of nationally standardized data for several contextual variables that may influence tuberculosis treatment outcomes, such as drug resistance, medication stockouts, quality of health programs, stigma, and migration patterns. Although migration was partially addressed through the variable “Proportion of new pulmonary TB cases among special population groups,” including immigrants as a population of interest, the lack of comprehensive and standardized measurements for the remaining factors at the municipal level precluded their inclusion in our analysis.

Another issue is that 4.14% of municipalities were excluded due to missing or unreported treatment closure data. While any data loss can theoretically introduce bias, this proportion is considered low and falls within acceptable limits for population-based epidemiological studies [53]. Moreover, no systematic pattern of missingness was identified that would suggest selection bias.

Finally, the study period encompassed the COVID-19 pandemic, which directly impacted TB indicators. WHO estimates indicate that 2020 and 2021 were characterized by reduced TB diagnoses and increased mortality. In Brazil, a slight decline in cure rates and an increase in treatment interruption were observed from 2020 onwards, reflecting the redirection of health service resources toward COVID-19 management at the expense of TB care [54,55,56]. This context represents a relevant factor that may have influenced the epidemiological dynamics of the disease during the study period.

## 5. Conclusions

Considering that variables from all three blocks were associated with treatment interruption, these findings suggest that socioeconomic, epidemiological, and healthcare access factors should be taken into account when formulating public policies and strategies to address TB treatment interruption. Public policies focused on providing continuous support, reducing social inequalities, and improving access to healthcare services may significantly reduce treatment interruption rates and improve public health indicators.

The heterogeneity of local contexts may influence outcomes; therefore, investigating the specific characteristics of each area can contribute to the development of strategies that reflect the realities of each territory.

## Figures and Tables

**Table 1:** Epidemiological variables related to the Tuberculosis Control Program, operational health indicators, and socioeconomic factors.

Epidemiological Variables of the Tuberculosis Control Program	Year	Source
Incidence rate of new pulmonary TB cases *	2018–022	DATASUS
TB mortality rate *	2018–2022	DATASUS
Proportion of sputum smear microscopy performed among new pulmonary TB cases *	2018–2022	DATASUS
Proportion of molecular rapid tests for TB performed among new pulmonary TB cases*	2018–2022	DATASUS
Proportion of drug susceptibility tests performed among new pulmonary TB cases*	2018–2022	DATASUS
Proportion of new pulmonary TB cases confirmed by laboratory tests *	2018–2022	DATASUS
Proportion of new pulmonary TB cases tested for HIV *	2018–2022	DATASUS
Proportion of TB-HIV coinfection among new pulmonary TB cases *	2018–2022	DATASUS
Proportion of new pulmonary TB cases among special population groups (incarcerated individuals, people experiencing homelessness, healthcare workers, immigrants, and Indigenous peoples) *	2018–2022	DATASUS
Proportion of contacts examined among new pulmonary TB cases *	2018–2022	DATASUS
Proportion of new pulmonary TB cases receiving Directly Observed Therapy (DOT) *	2018–2022	DATASUS
AIDS case detection rate	2019	DATHI
**Variables related to health service coverage**		
Number of SUS hospital beds per 1000 inhabitants	2019	DATASUS
Number of medical consultations per inhabitant per year	2019	DATASUS
Percentage of homogeneity in vaccine coverage across vaccines	2019	DATASUS
Coverage rate of the Family Health Strategy (FHS)	2019	Site e-GESTOR
Coverage rate of Community Health Agents (CHA)	2019	Site e-GESTOR
**Socioeconomic variables**		
Resident population	2022	IBGE
Population density	2022	IBGE
Municipal Human Development Index (MHDI)	2010	IBGE
Household per capita income	2010	Atlas Brasil
Gross Domestic Product (GDP) per capita	2021	IBGE
Gini Index	2010	Atlas Brasil
Unemployment rate	2010	Atlas Brasil
Illiteracy rate (≥15 years old)	2022	IBGE
Household crowding	2010	Atlas Brasil
Life expectancy at birth	2010	Atlas Brasil
Infant mortality rate	2019	DATASUS
Proportion of the population living in poverty	2010	Atlas Brasil
Proportion of the population residing in rural areas	2022	IBGE

Note: * The variables related to the TB Control Program used aggregate data from 2018 to 2022.

**Table 2:** Socioeconomic, Epidemiological, and Health System Operational Characteristics of Brazilian Municipalities, 2018–2022 *.

Variable	Mean (SD)	Median (25th–75th Percentile IQR)
Treatment Interruption Rate (2018–2022)	8.0 (13.96)	0.00 (0.00–12.00)
**Epidemiological and Operational Variables Related to the Tuberculosis Control Program**		
Incidence rate of new pulmonary TB cases	92.251 (129.422)	67.760 (40.040–107.840)
TB mortality rate	8.37 (10.54)	5.83 (0.00–12.97)
Proportion of new pulmonary TB cases undergoing sputum smear microscopy	76.27 (24.23)	81.25 (66.67–100.00)
Proportion of new pulmonary TB cases tested with rapid molecular TB tests	27.16 (29.36)	16.77 (0.00–45.45)
Proportion of drug susceptibility tests performed among new pulmonary TB cases (2019)	8.69 (17.59)	0.00 (0.00–9.38)
Proportion of new pulmonary TB cases confirmed by laboratory tests	69.65 (25.30)	73.53 (55.56–88.89)
Proportion of new pulmonary TB cases tested for HIV	79.08 (25.53)	87.68 (66.67–100.00)
Proportion of TB-HIV coinfection among new pulmonary TB cases	5.87 (12.06)	0.00 (0.00–7.89)
Proportion of new pulmonary TB cases in special populations (incarcerated individuals, people experiencing homelessness, healthcare workers, immigrants, and Indigenous peoples)	10.11 (20.01)	0.00 (0.00–12.50)
Proportion of contacts examined among new pulmonary TB cases	80.20 (54.55)	88.89 (63.89–100.00)
Proportion of new pulmonary TB cases receiving Directly Observed Therapy (DOT)	47.87 (35.03)	50.00 (14.29–77.78)
AIDS case detection rate	9.79 (12.28)	6.44 (0.00–15.46)
**Variables Related to Health Service Coverage**		
Number of SUS hospital beds per 1000 inhabitants	1.37 (1.62)	1.11 (0.00–2.01)
Number of medical consultations per inhabitant per year	6.38 (5.39)	4.90 (2.67–8.67)
Homogeneity in vaccine coverage across vaccines (%)	35.10 (30.05)	30.00 (10.00–60.00)
Coverage rate of the Family Health Strategy (FHS)	88.25 (22.01)	100.00 (84.99–100.00)
Coverage rate of Community Health Agents (CHA)	90.23 (21.36)	100.00 (96.30–100.00)
**Socioeconomic Variables**		
Resident population (IBGE)	40,091 (217,521)	13,016 (6347–26,881)
Population density (inhabitants/km^2^)	126.89 (626.59)	25.82 (11.93–60.17)
Gini Index	0.50 (0.07)	0.50 (0.45–0.54)
Municipal Human Development Index (MHDI)	0.658 (0.073)	0.664 (0.597–0.718)
Per capita Gross Domestic Product (GDP, R$)	33,458.27 (42,955.50)	22,951.75 (12,588.25–39,829.41)
Household per capita income (R$)	490.82 (245.07)	461·47 (276.94–651.41)
Unemployment rate	6.96 (3.76)	6.46 (4.41–8.78)
Household crowding	25.93 (13.08)	24.01 (16.09–33.33)
Life expectancy at birth	73.04 (2.70)	73.40 (71.08–75.14)
Infant mortality rate	25.98 (23.90)	23.05 (10.36–35.71)
Illiteracy rate (≥15 years old)	11.11 (7.06)	9.14 (5.09–16.72)
Proportion of the population living in poverty	23.66 (18.11)	18.97 (7.07–39.28)
Proportion of the population residing in rural areas	29.83(19.98)	27.44(12.97–44.49)

Source: Prepared by the authors using data from the following databases: DATASUS, DATHI, e-Gestor APS, IBGE, and Atlas Brasil. SD: standard deviation; IQR: interquartile range (25th–75th percentiles); TB: tuberculosis; HIV: human immunodeficiency virus; SUS: Brazilian Unified Health System; IBGE: Brazilian Institute of Geography and Statistics; MHDI: Municipal Human Development Index; GDP: gross domestic product. * Data subject to revision.

**Table 3:** Crude analysis of the relationship between epidemiological, operational, health service coverage, and socioeconomic variables of municipalities and the percentage of pulmonary tuberculosis treatment interruption in Brazil, 2018–2022 *.

Variables (Proportion and Zero-Inflated)	Crude Analysis	
	Coef. B	*p*-Value
**Epidemiological and Operational Variables Related to the Tuberculosis Control Program**		
Incidence rate of new pulmonary TB cases		
Proportion	−0.002	<0.001
Zero-inflated	−0.015	<0.001
TB mortality rate		
Proportion	−0.012	<0.001
Zero-inflated	−0.025	<0.001
Proportion of sputum smear microscopy performed among new pulmonary TB cases		
Proportion	−0.005	<0.001
Zero-inflated	0.0005	0.692
Proportion of molecular rapid tests for TB performed among new pulmonary TB cases		
Proportion	−0.002	0.006
Zero-inflated	0.001	0.581
Proportion of drug susceptibility tests performed among new pulmonary TB cases		
Proportion	−0.003	0.052
Zero-inflated	−0.004	0.022
Proportion of new pulmonary TB cases confirmed by laboratory tests		
Proportion	−0.001	0.655
Zero-inflated	−0.004	<0.001
Proportion of new pulmonary TB cases tested for HIV		
Proportion	−0.011	<0.001
Zero-inflated	−0.006	<0.001
Proportion of TB-HIV coinfection among new pulmonary TB cases		
Proportion	0.011	<0.001
Zero-inflated	−0.008	0.001
Proportion of new pulmonary TB cases among special population groups (incarcerated individuals, people experiencing homelessness, healthcare workers, immigrants, and Indigenous peoples)		
Proportion	−0.004	0.004
Zero-inflated	−0.013	<0.001
Proportion of contacts examined among new pulmonary TB cases		
Proportion	−0.004	<0.001
Zero-inflated	0.001	0.127
Proportion of new pulmonary TB cases receiving Directly Observed Therapy (DOT)		
Proportion	−0.006	<0.001
Zero-inflated	0.010	<0.001
AIDS case detection rate		
Proportion	−0.010	<0.001
Zero-inflated	−0.040	<0.001
**Variables Related to Health Service Coverage**		
Number of SUS hospital beds per 1000 inhabitants		
Proportion	−0.014	0.402
Zero-inflated	−0.039	0.025
Number of medical consultations per inhabitant per year		
Proportion	0.019	<0.001
Zero-inflated	0.033	<0.001
Percentage of homogeneity in vaccine coverage across vaccines		
Proportion	0.003	<0.001
Zero-inflated	0.011	<0.001
Coverage rate of the Family Health Strategy (FHS)		
Proportion	0.003	0.003
Zero-inflated	0.027	<0.001
Coverage rate of Community Health Agents (CHA)		
Proportion	0.002	0.023
Zero-inflated	0.027	<0.001
**Socioeconomic Variables**		
Resident population (IBGE)		
Proportion	−0.0000001	0.987
Zero-inflated	−0.00008	<0.001
Population density (inhabitants/km^2^)		
Proportion	−0.00003	0.504
Zero-inflated	−0.009	<0.001
Gini Index		
Proportion	−2.302	<0.001
Zero-inflated	−4.369	<0.001
Municipal Human Development Index (MHDI)		
Proportion	0.585	0.030
Zero-inflated	−3.258	<0.001
Gross Domestic Product (GDP) per capita (R$)		
Proportion	0.0000007	0.916
Zero-inflated	−0.000001	0.741
Household per capita income (R$)		
Proportion	0.0001	0.091
Zero-inflated	−0.001	<0.001
Unemployment rate		
Proportion	−0.043	<0.001
Zero-inflated	−0.086	<0.001
Household crowding		
Proportion	−0.013	<0.001
Zero-inflated	−0.032	<0.001
Life expectancy at birth		
Proportion	0.028	<0.001
Zero-inflated	−0.057	<0.001
Infant mortality rate		
Proportion	0.001	0.299
Zero-inflated	0.0001	0.929
Illiteracy rate (≥15 years old)		
Proportion	−0.009	0.003
Zero-inflated	0.040	<0.001
Proportion of the population living in poverty		
Proportion	−0.005	<0.001
Zero-inflated	0.005	<0.001
Proportion of the population residing in rural areas		
Proportion	0.005	<0.001
Zero-inflated	0.028	<0.001

Source: Prepared by the authors using data from the following databases: DATASUS, DATHI, e-Gestor APS, IBGE, and Atlas Brasil. Coef. B: beta coefficient; TB: tuberculosis; AIDS: acquired immunodeficiency syndrome (Portuguese acronym); HIV: human immunodeficiency virus; SUS: Brazilian Unified Health System; IBGE: Brazilian Institute of Geography and Statistics; MHDI: Municipal Human Development Index; GDP: gross domestic product. Data are subject to revision. Notes: numerical values of the beta coefficient and *p*-values have been rounded. * Data subject to revision.

**Table 4:** Adjusted analysis of the relationship between epidemiological, operational, health service coverage, and socioeconomic variables of municipalities and the percentage of pulmonary tuberculosis treatment interruption in Brazil, 2018–2022 *.

Variables (Proportion and Zero-Inflated)	Adjusted Analysis							
	Model 1—TB Variables		Model 2—TB + Health Variables		Model 3—TB + Health + Sociodemographic Variables		Model 4—TB + Health + Sociodemographic + Geographic Variables	
	Coef. B	*p*-Value	Coef. B	*p*-Value	Coef. B	*p*-Value	Coef. B	*p*-Value
**Epidemiological and Operational Variables Related to the Tuberculosis Control Program**								
Incidence rate of new pulmonary TB cases								
Proportion	−0.002	<0.001	−0.006	<0.001	−0.001	<0.001	−0.001	<0.001
Zero-inflated	−0.014	<0.001	−0.013	<0.001	−0.010	<0.001	−0.010	<0.001
Proportion of sputum smear microscopy performed among new pulmonary TB cases								
Proportion	−0.011	<0.001	−0.011	<0.001	−0.011	<0.001	−0.011	<0.001
Zero-inflated	-	-	-	-	-	-	-	-
Proportion of molecular rapid tests for TB performed among new pulmonary TB cases								
Proportion	−0.008	<0.001	−0.008	<0.001	−0.008	<0.001	−0.009	<0.001
Zero-inflated	-	-	-	-	-	-	-	-
Proportion of drug susceptibility tests performed among new pulmonary TB cases								
Proportion	0.005	0.002	0.005	0.001	0.003	0.027	0.003	0.078
Zero-inflated	-	-	-	-	-	-	-	-
Proportion of new pulmonary TB cases confirmed by laboratory tests								
Proportion	0.012	<0.001	0.012	<0.001	0.013	<0.001	0.014	<0.001
Zero-inflated	−0.004	0.006	−0.003	0.024	-	-	-	-
Proportion of new pulmonary TB cases tested for HIV								
Proportion	-	-	-	-	-	-	-	-
Zero-inflated	−0.003	0.048	-	-	-	-	-	-
Proportion of TB-HIV coinfection among new pulmonary TB cases								
Proportion	-	-	-	-	-	-	-	-
Zero-inflated	−0.007	0.017	−0.008	0.009	-	-	-	-
Proportion of new pulmonary TB cases among special population groups (incarcerated individuals, people experiencing homelessness, healthcare professionals, immigrants, and Indigenous peoples)								
Proportion	-	-	-	-	-	-	-	-
Zero-inflated	−0.003	0.092	-	-	-	-	-	-
Proportion of contacts examined among new pulmonary TB cases								
Proportion	−0.003	<0.001	−0.003	<0.001	−0.003	< 0.001	−0.003	<0.001
Zero-inflated	-	-	-	-	-	-	-	-
Proportion of new pulmonary TB cases receiving Directly Observed Therapy (DOT)								
Proportion	−0.004	<0.001	−0.005	<0.001	−0.005	<0.001	−0.005	<0.001
Zero-inflated	0.012	<0.001	0.009	<0.001	0.010	<0.001	0.010	<0.001
AIDS case detection rate								
Proportion	−0.006	0.001	−0.004	0.011	−0.004	0.049	−0.004	0.007
Zero-inflated	−0.023	<0.001	−0.022	<0.001	−0.006	0.040	−0.006	0.039
**Variables Related to Health Service Coverage**								
Number of SUS hospital beds per 1000 inhabitants								
Proportion	-	-	-	-	-	-	-	-
Zero-inflated	-	-	−0.067	0.001	-	-	-	-
Number of medical consultations per inhabitant per year								
Proportion	-	-	0.012	0.004	-	-	-	-
Zero-inflated	-	-	0.055	<0.001	0.043	<0.001	0.043	<0.001
Percentage of homogeneity in vaccine coverage across vaccines								
Proportion	-	-	0.002	0.013	-	-	-	-
Zero-inflated	-	-	0.005	<0.001	-	-	-	-
Coverage rate of the Family Health Strategy (FHS)								
Proportion	-	-	0.005	<0.001	0.004	<0.001	0.004	<0.001
Zero-inflated	-	-	0.025	<0.001	-	-	-	-
**Socioeconomic Variables**								
Resident population (IBGE)								
Proportion	-	-	-	-	-	-	-	-
Zero-inflated	-	-	-	-	−0.00007	<0.001	−0.00007	<0.001
Gini Index								
Proportion	-	-	-	-	−1.784	<0.001	−1.851	<0.001
Zero-inflated	-	-	-	-	-	-	-	-
Household crowding								
Proportion	-	-	-	-	−0.007	<0.001	−0.008	<0.001
Zero-inflated	-	-	-	-	−0.019	<0.001	−0.019	<0.001
Illiteracy rate (≥15 years)								
Proportion	-	-	-	-	−0.018	<0.001	−0.011	0.028
Zero-inflated	-	-	-	-	0.036	<0.001	0.038	<0.001
Proportion of the population residing in rural areas								
Proportion	-	-	-	-	0.009	<0.001	0.010	<0.001
Zero-inflated	-	-	-	-	0.008	<0.001	0.008	<0.001
***Geographic Variables***								
pb(X)								
Proportion	-	-	-	-	-	-	−0.017	<0.001
Zero-inflated	-	-	-	-	-	-	−0.0008	0.912
pb(Y)								
Proportion	-	-	-	-	-	-	0.0005	0.891
Zero-inflated	-	-	-	-	-	-	−0.003	0.729
Deviance	2555.469		2224.58		1158.533		1035.138	
AIC	2591.469		2270.58		1206.533		1122.579	
BIC	2708.04		2419.441		1361.876		1405.562	

Source: Prepared by the authors using data from DATASUS, DATHI, e-Gestor APS, IBGE, and Atlas Brasil. Coef. B: beta coefficient; TB: tuberculosis; HIV: human immunodeficiency virus; AIDS: acquired immunodeficiency syndrome (Portuguese acronym); SUS: Unified Health System; IBGE: Brazilian Institute of Geography and Statistics; AIC: Akaike Information Criterion; BIC: Bayesian Information Criterion. * Data subject to revision. Notes: numerical values of the beta coefficient and *p*-values have been rounded.

## Data Availability

The raw data supporting the conclusions of this article will be made available by the authors on request.

## References

[1] Organização Mundial da Saúde (2024). Relatório Global Sobre Tuberculose: 2024.

[2] Organização Mundial da Saúde (2020). Relatório Global Sobre Tuberculose 2020.

[3] Departamento de Doenças de Condições Crônicas e Infecções Sexualmente Transmissíveis, Secretaria de Vigilância em Saúde, Ministério da Saúde (2019). Manual de Recomendações para o Controle da Tuberculose no Brasil.

[4] Departamento de HIV/AIDS, Tuberculose, Hepatites Virais e Infecções Sexualmente Transmissíveis, Secretaria de Vigilância em Saúde e Ambiente, Ministério da Saúde (2024). Nota Informativa no 5/2024-CGTM/.DATHI/SVSA/MS. Implementação do tratamento encurtado datuberculose sensível não grave em crianças eadolescentes (2RHZ (E)/ 2RH).

[5] Departamento de HIV, Aids, Tuberculose, Hepatites Virais e Infecções Sexualmente Transmissíveis, Secretaria de Vigilância em Saúde e Ambiente, Ministério da Saúde (2025). Boletim Epidemiológico: Tuberculose 2025.

[6] Departamento de Doenças de Condições Crônicas e Infecções Sexualmente Transmissíveis, Secretaria de Vigilância em Saúde, Ministério da Saúde (2017). Brasil Livre da Tuberculose: Plano Nacional pelo Fim da Tuberculose como Problema de Saúde Pública.

[7] Organização Mundial da Saúde (2016). Relatório global sobre tuberculose de 2016.

[8] Harling G., Neto A.S.L., Sousa G.S., Machado M.M.T., Castro M.C. (2017). Determinantes da transmissão da tuberculose e abandono do tratamento em Fortaleza, Brasil. BMC Saúde Pública.

[9] Pelissari D.M., Rocha M.S., Bartholomay P., Sanchez M.N., Duarte E.C., Arakaki-Sanchez D., Dantas C.O., Jacobs M.G., Andrade K.B., Codenotti S.B. (2018). Identificando cenários socioeconômicos, epidemiológicos e operacionais para o controle da tuberculose no Brasil: Um estudo ecológico. BMJ Open.

[10] Pelissari D.M., Diaz-Quijano F.A. (2017). Aglomeração domiciliar como potencial mediador dos determinantes socioeconômicos da incidência de tuberculose no Brasil. PLoS ONE.

[11] Rigby R.A., Stasinopoulos D.M. (2005). Generalized additive models for location, scale and shape (with discussion). J. R. Stat. Soc. Ser. C Appl. Stat..

[12] Conselho Nacional de Saúde (2016). Resolução n.º 510, de 07 de abril de 2016. https://www.gov.br/conselho-nacional-de-saude/pt-br/acesso-a-informacao/legislacao/resolucoes/2016/resolucao-no-510.pdf/view.

[13] Cortez A.O., Melo A.C., Neves L.O., Resende K.A., Camargos P. (2021). Tuberculose no Brasil: Um país, múltiplas realidades. J Bras Pneumol..

[14] San Pedro A., Oliveira R.M. (2013). Tuberculosis and socioeconomic indicators: Systematic review of the literature. Rev. Panam. Salud Publica.

[15] Giacomet C.L., Ramos A.C., Moura H.S., Berra T.Z., Alves Y.M., Delpino F.M., Farley J.E., Reynolds N.R., Alonso J.B., Teibo T.K. (2023). A distributional regression approach to modeling the impact of structural and intermediary social determinants on communities burdened by tuberculosis in Eastern Amazonia–Brazil. Arch. Public Health..

[16] Maciel E.L., Reis-Santos B. (2015). Determinants of tuberculosis in Brazil: From conceptual framework to practical application. Rev. Panam. Salud Publica..

[17] Prado Junior J.C., Medronho R.d. (2021). Análise espacial da cura da tuberculose na atenção primária no Rio de Janeiro, Brasil. BMC Saúde Pública.

[18] Emani S., Alves K., Alves L.C., da Silva D.A., Oliveira P.B., Castro M.C., Cohen T., Couto R.D., Sanchez M., Menzies N.A. (2024). Quantifying gaps in the tuberculosis care cascade in Brazil: A mathematical model study using national program data. PLoS Medicine..

[19] Oliveira GP, Torrens AW, Bartholomay P, Barreira D (2013). Tuberculosis in Brazil: Last ten years analysis—2001–2010. Braz. J. Infect. Dis..

[20] Sousa G.J.B., Maranhão T.A., Leitão T.M.J.S., Souza J.T., Moreira T.M.M., Pereira M.L.D. (2021). Prevalência e fatores associados ao abandono do tratamento da tuberculose. Rev. Esc. Enferm. USP.

[21] Alves K.K.A.F., Borralho L.M., Araújo A.J., Bernardino I.M., Figueiredo T.M.R.M. (2020). Fatores associados à recuperação e ao abandono do tratamento da tuberculose na população carcerária. Revista Brasileira de Epidemiologia.

[22] Creswell J., Rai B., Wali R., Sudrungrot S., Adhikari L.M., Pant R., Pyakurel S., Uranw D., Codlin A.J. (2015). Introducing new tuberculosis diagnostics: The impact of Xpert^®^ MTB/RIF testing on case notifications in Nepal. Int. J. Tuberc. Lung Dis..

[23] Albert H., Nathavitharana R.R., Isaacs C., Pai M., Denkinger C.M., Boehme C.C. (2016). Development, roll-out and impact of Xpert MTB/RIF for tuberculosis: What lessons have we learnt and how can we do better?. Eur. Respir. J..

[24] Storla D.G., Yimer S., Bjune G.A. (2008). A systematic review of delay in the diagnosis and treatment of tuberculosis. BMC Public Health.

[25] Creswell J., Codlin A.J., Andre E., Micek M.A., Bedru A., Carter E.J., Yadav R.P., Mosneaga A., Rai B., Banu S. (2014). Results from early programmatic implementation of Xpert MTB/RIF testing in nine countries. BMC Infect Dis..

[26] Fundação Oswaldo Cruz (2022). Laboratório de Referência Nacional do Hélio Fraga Capacita Monitores em Teste Rápido Molecular Para Tuberculose.

[27] Maciel E.L.N., Sales C.M.M., Bertolde A.I., Reis-Santos B., Fregona G., Zandonade E. (2015). Implementação do teste rápido molecular para tuberculose em unidades de saúde: Desafios e perspectivas no Brasil. BMC Health Serv Res..

[28] Baluku J.B., Kabamooli R. (2021). A; Kajumba, N; Nabwana, M,; Kateete, D; Kiguli, S; Andia-Biraro, I. Contact tracing is associated with treatment success of index tuberculosis cases in Uganda. Int. J. Infect. Dis..

[29] Matias G.L., Sales M.V., Andrade G.S., Teixeira B.D., Tenorio M.E., Palácio M.A., Correia M.L., Takenami I. (2024). Diagnosis and treatment of latent tuberculosis infection among household contacts in inland Bahia, Brazil: A cross-sectional follow-up study. Sao Paulo Med J..

[30] Lima L.V., Pavinati G., Palmieri I.G.S., Vieira J.P., Blasque J.C., Higarashi I.H., Fernandes C.A.M., Magnabosco G.T. (2023). Fatores associados à perda de seguimento no tratamento da tuberculose no Brasil: Um estudo de coorte retrospectivo. Rev. Gaúcha Enferm..

[31] Cola J.P., Pinto A.S., Souza J.S., Hertel J.F.H.F., Galavote H.S., do Prado T.N., Maciel E.L.N. (2024). Fatores associados ao abandono do tratamento da tuberculose: Um estudo transversal entre 2014 e 2019. J. Hum. Growth Dev..

[32] Santos D.A.S., Marques A.L.A., Goulart L.S., Mattos M., Olinda R.A. (2021). Fatores associados ao abandono do tratamento da tuberculose pulmonar. Cogitare Enferm..

[33] Perlaza C.L., Mosquera F.E.C., Murillo L.M.R., Sepulveda V.B., Arenas C.D.C. (2023). Fatores de abandono do tratamento da tuberculose na rede pública de saúde. Rev Saúde Pública.

[34] Ferreira M.R.L., Bonfim R.O., Siqueira T.C., Orfão N.H. (2018). Abandono do tratamento da tuberculose: Uma revisão integrativa. Rev. Enferm. Contemp..

[35] Departamento de HIV/Aids, Tuberculose, Hepatites Virais e Infecções Sexualmente Transmissíveis, Secretaria de Vigilância em Saúde e Ambiente, Ministério da Saúde (2023). Boletim Epidemiológico: Coinfecção TB-HIV 2022.

[36] Quintero M.C.F., Vendramini S.H.F., Santos M.L.S.G., Rocha dos Santos M., Gazetta C.E., Garcia Lourenção L., Sperli Geraldes Soler Z.A., da Cruz Oliveira S.A., Geraldes Marin dos Santos Sasaki N., Zanon Ponce M.A. (2018). Acesso ao diagnóstico de tuberculose em municípios brasileiros de médio porte. Rev. Salud Pública.

[37] Lucena L.A., Dantas G.B.S., Carneiro T.V., Lacerda H.G. (2023). Fatores Associados ao Abandono do Tratamento da Tuberculose no Brasil: Uma Revisão Sistemática. Rev. Soc. Bras. Med. Trop..

[38] Souza M.S.P.L., Aquino R., Pereira S.M., Costa M.D.C.N., Barreto M.L., Natividade M., Ximenes R., Souza W., Dantas O.M., Braga J.U. (2015). Fatores associados ao acesso geográfico aos serviços de saúde para pessoas com Tuberculose em três capitais do Nordeste brasileiro. Cad. Saúde Pública.

[39] Fonseca E.P., Melo L.B., Pereira A.C., Mendes K.L.C., Verdi M.R.D.M., Meneghim M.C. (2023). Não Adesão ao Tratamento da Tuberculose no Estado de São Paulo: Reflexões sobre Gestão em Saúde e Enfermagem. https://preprints.scielo.org/index.php/scielo/preprint/view/7106/version/7517.

[40] Ferreira H.S., Mascarello K.C., Cola J.P., Vieira A.C.B.C., Carlesso G.F., Sales C.M.M., Maciel E.L.N. (2022). Fatores sociais preditores de cura da Tuberculose em capitais brasileiras. Rev. Bras. Pesq. Saúde..

[41] Mutembo S., Mutanga J.N., Musokotwane K., Kanene C., Dobbin K., Yao X., Li C., Marconi V.C., Whalen C.C. (2019). Urban-rural disparities in treatment outcomes among recurrent TB cases in Southern Province, Zambia. BMC Infect. Dis..

[42] Ahmed M., Mohan R. (2021). A comparative study of factors for interruption of antitubercular treatment among defaulters in urban and rural areas of Kamrup District, Assam. J. Family Med. Primary Care.

[43] Jesus G.S., Pescarini J.M., Silva A.F., Torrens A., Carvalho W.M., Junior E.P., Ichihara M.Y., Barreto M.L., Rebouças P., Macinko J. (2022). The effect of primary health care on tuberculosis in a nationwide cohort of 7· 3 million Brazilian people: A quasi-experimental study. Lancet Global Health.

[44] Pinto F.G., Garcia W.M.B., Silva-Junior R.G.P., Ferro G.B., da Costa A.G., de Carvalho Zavarise M., da Silva Morais C.A., Mendes E.A.R., Gaia S.L., Lobato M.Y.F. (2022). Adesão ao tratamento da tuberculose na Atenção Primária à Saúde: Fatores favoráveis e desfavoráveis para esse processo. Res. Soc. Dev..

[45] Lima S.V.M.A., Araújo K.C.G.M., Nunes M.A.P., Nunes C. (2021). Identificação precoce de indivíduos em risco de perda de seguimento do tratamento da tuberculose: Uma análise hierárquica generalizada. Heliyon.

[46] Aragão F.B.A., Calori M.Y., Berra T.Z., Ramos A.C.V., Maciel E.L.N., Cunha J.H.D.S., Souza L.B.D., Santos Neto M., Arcêncio R.A., Fiorati R.C. (2024). Proteção social em áreas vulneráveis à tuberculose: Um estudo de métodos mistos em São Luís, Maranhão. Rev. Brás Enferm..

[47] Orlandi G.M., Pereira E.G., Biagolini R.E.M., França F.O.S., Bertolozzi M.R. (2019). Incentivos sociais para adesão ao tratamento da tuberculose. Rev. Brás Enferm..

[48] Furlan M.C.R., Oliveira SPde Marcon S.S. (2012). Fatores associados ao abandono do tratamento de tuberculose no estado do Paraná. Acta Paul. Enferm..

[49] Rocha G.S.S., Lima M.G., Moreira J.L., Ribeiro K.C., Ceccato M.D.G.B., Carvalho W.D.S., Silveira M.R. (2015). Conhecimento dos agentes comunitários de saúde sobre a tuberculose, suas medidas de controle e tratamento diretamente distribuído. Cad. Saúde Pública..

[50] Vieira-Meyer A.P.G.F., Morais A.P.P., Campelo I.L.B., Guimarães J.M.X. (2025). Violência e vulnerabilidade no território do agente comunitário de saúde: Implicações no enfrentamento da COVID-19. Ciên. Saúde Colet..

[51] Morgenstern H. (1995). Ecologic studies in epidemiology: Concepts, principles, and methods. Annu. Rev. Public. Health.

[52] Diez-Roux A.V. (2000). Multilevel analysis in public health research. Annu. Rev. Public. Health.

[53] Rothman K.J., Greenland S., Lash T.L. (2008). Modern Epidemiology.

[54] Hino P., Yamamoto T.T., Magnabosco G.T., Bertolozzi M.R., Taminato M., Fornari L.F. (2021). Impacto da COVID-19 no controle e reorganização da atenção à tuberculose. Acta Paul. Enferm..

[55] Ribeiro R.R., Andrade R.L.D.P., Silva DCda Sthal H.C., Oliveira J.A., Regis I.M., Gonzalez R.I.C. (2025). Repercussão da pandemia da COVID-19 nas ações de controle da tuberculose na atenção primária à saúde: Revisão de escopo. Ciênc. Saúde Coletiva.

[56] Silva JAda Rufino E.N.M., Sampaio B.F., Silva D.M. (2023). Impacto da pandemia de covid-19 no número de casos e na mortalidade da tuberculose. Rev. Ibero-Am. De Humanidades Ciências E Educ..

